# A case series exploring the human milk polyclonal IgA1 response to repeated SARS-CoV-2 vaccinations by LC–MS based fab profiling

**DOI:** 10.3389/fnut.2023.1305086

**Published:** 2024-01-15

**Authors:** Sebastiaan C. de Graaf, Albert Bondt, Danique M. H. van Rijswijck, Hannah G. Juncker, Sien J. Mulleners, Mirjam J. A. Damen, Max Hoek, Britt J. van Keulen, Johannes B. van Goudoever, Albert J. R. Heck, Kelly A. Dingess

**Affiliations:** ^1^Biomolecular Mass Spectrometry and Proteomics, Bijvoet Center for Biomolecular Research and Utrecht Institute for Pharmaceutical Sciences, University of Utrecht, Utrecht, Netherlands; ^2^Netherlands Proteomics Center, Utrecht, Netherlands; ^3^Department of Pediatrics, Amsterdam Reproduction and Development Research Institute, Emma Children’s Hospital, Amsterdam UMC, University of Amsterdam and Vrije Universiteit Amsterdam, Amsterdam, Netherlands; ^4^Swammerdam Institute for Life Sciences, Center for Neuroscience, University of Amsterdam, Amsterdam, Netherlands

**Keywords:** antigen binding fragment, immunoglobulin A, mass spectrometry, human milk, COVID-19

## Abstract

**Introduction:**

Upon vaccination against severe acute respiratory syndrome coronavirus 2 (SARS-CoV-2) humans will start to produce antibodies targeting virus specific antigens that will end up in circulation. In lactating women such antibodies will also end up in breastmilk, primarily in the form of secretory immunoglobulin A1 (SIgA1), the most abundant immunoglobulin (Ig) in human milk. Here we set out to investigate the SIgA1 clonal repertoire response to repeated SARS-CoV-2 vaccination, using a LC–MS fragment antigen-binding (Fab) clonal profiling approach.

**Methods:**

We analyzed the breastmilk of six donors from a larger cohort of 109 lactating mothers who received one of three commonly used SARS-CoV-2 vaccines. We quantitatively monitored the SIgA1 Fab clonal profile over 16 timepoints, from just prior to the first vaccination until 15  days after the second vaccination.

**Results:**

In all donors, we detected a population of 89–191 vaccine induced clones. These populations were unique to each donor and heterogeneous with respect to individual clonal concentrations, total clonal titer, and population size. The vaccine induced clones were dominated by persistent clones (68%) which came up after the first vaccination and were retained or reoccurred after the second vaccination. However, we also observe transient SIgA1 clones (16%) which dissipated before the second vaccination, and vaccine induced clones which uniquely emerged only after the second vaccination (16%). These distinct populations were observed in all analyzed donors, regardless of the administered vaccine.

**Discussion:**

Our findings suggest that while individual donors have highly unique human milk SIgA1 clonal profiles and a highly personalized SIgA1 response to SARS-CoV-2 vaccination, there are also commonalities in vaccine induced responses.

## Introduction

Immunoglobulins (Ig), or antibodies, are a key part of the adaptive immune response capable of specifically recognizing and binding to antigens derived from bacteria or viruses initiating and aiding in their neutralization. Every individual has a unique antibody repertoire generated by a magnitude of distinct antibody-producing B cells, with estimates ranging from 10^13^ to 10^18^ (1, 2). Throughout our lives these repertoires are built up by encountering a huge variety of pathogens and other foreign stimuli, which we are exposed to daily or at specific moments in time, such as vaccines. However, at a given moment in time there are likely only hundreds to thousands of different detectable antibodies in human serum and milk, and typically the top 50 most abundant Ig clones account for up to 90% of the complete Ig repertoire (3–5).

In our first moments of life, we begin to build this repertoire and are provided passive immunity through breastfeeding, receiving in most cases our mother’s own unique antibodies. After natural infection with severe acute respiratory syndrome coronavirus 2 (SARS-CoV-2), SARS-CoV-2 specific antibodies with neutralizing capacity are present in human milk and are thought to provide immunity to infants (6–12). Due to the overwhelming health benefits of breastfeeding and the absence of vertical transmission of SARS-CoV-2 via human milk (6, 8, 13–15) have led to the advice of the WHO to encourage mothers to continue breastfeeding their infant during the COVID-19 pandemic (16). Recently, several SARS-CoV-2 vaccines have been widely administered to people around the world. While the accumulated evidence has shown that these vaccines are safe and effective also for pregnant and lactating women (17–22), this more vulnerable group was excluded from initial SARS-CoV-2 vaccine trials. Therefore, information regarding vaccine driven antibody development in lactating women is still rather limited. This information is beneficial for breastfeeding women to make a well-informed decision regarding vaccination to confer protection to not only themselves, but also their immune naïve infant (23). The most abundant Ig in human milk is IgA at a concentration of 1.0–2.6 g/L being 10 to 100 times greater than IgG and IgM, respectively, (24, 25). IgA comes in two subclasses IgA1 and IgA2, with IgA1 typically being the more abundant subclass in human milk. We recently developed methods to study IgA1 clonal repertoires in human serum and milk. After affinity-purification, all IgA (IgA1 and IgA2) molecules from human serum or milk (4, 5) become bound to the affinity resins, whereafter we use specific enzymes to cleave IgA1 molecules selectively, yielding the fragment antigen binding (Fab) domains that harbor the complementarity determining regions. These Fabs are then subjected to intact mass analysis by LC–MS clonal profiling. This yields a clonal profile that typically contains several hundred unique clones, each identified by a specific LC–MS signature based on mass and retention time. We can quantify the human milk concentrations of each Fab clone by spiking in recombinant IgA1 mAb standards (4), enabling us to monitor the abundance of individual clones over time. Monitoring the human milk secretory IgA1 (SIgA1) clonal repertoire of healthy individuals, we observed that they are relatively simple, being dominated by just a few hundred to thousand different clones at a given time. These repertoires are unique and highly personalized as we do not observe the same clones in more than one donor. Furthermore, we found the human milk SIgA1 repertoires of healthy donors to be very stable over time, with all SIgA1 Fab clones having Pearson correlations >0.8 (4). However, the clonal repertoires of individuals that experience serious illness, can undergo distinct and sudden changes (3, 26).

Mothers that were previously infected with SARS-CoV-2 have significantly higher concentrations of spike specific IgA in their breastmilk than negative controls (9), and using LC–MS we were able to detect spike specific SIgA1 Fab fragments in these donors. Interestingly, concentrations of spike specific IgA in human milk had little correlation with neutralization capability, and spike specific SIgA1 Fabs were of a relatively low concentration when compared to total SIgA1 in human milk. Other studies have also shown weak correlations between antibody titers and the frequency of recirculating memory B cells relative to a respective antigen (27). These findings suggest that high concentrations of antibodies may not be good predictors for effective viral recognition and binding. Detailed knowledge about the emergence and evolution of antibodies in response to vaccination could render better insights into the immunity they provide and thereby yield better predictors for its longevity and effectivity.

Here, we aim to expand the knowledge about the antibody response of lactating women following SARS-CoV-2 vaccination by investigating the SIgA1 profiles of six individuals that received repeated mRNA-based or vector-based SARS-CoV-2 vaccines. Donors and their samples for this observational longitudinal case series were selected from a previously described cohort, with the criteria of having high SARS-CoV-2-specific IgA titers in human milk (28). Using LC–MS Fab clonal profiling, we monitored the abundance of individual SIgA1 clones and studied the antibody response at a clonal level of detail. We used a novel computational approach in this study to detect SIgA1 Fab clonal populations emerging after vaccination by eliminating all clones that were present before a response to vaccination could be expected. The human milk SIgA1 clonal repertoires of six individual donors receiving one of three SARS-CoV-2 vaccines were quantitatively monitored over 16 timepoints. All six donors had unique SIgA1 clonal repertoires in which longitudinal changes were observed, with novel clonal populations emerging after both the initial and second vaccination. Our data reveals that antibody responses to vaccination are highly personalized traits and argues for monitoring antibody responses beyond the total Ig titer level, using a more detailed, personalized, and longitudinal approach.

## Results

### Vaccination results in a heterogeneous polyclonal response

**Figure 1:**
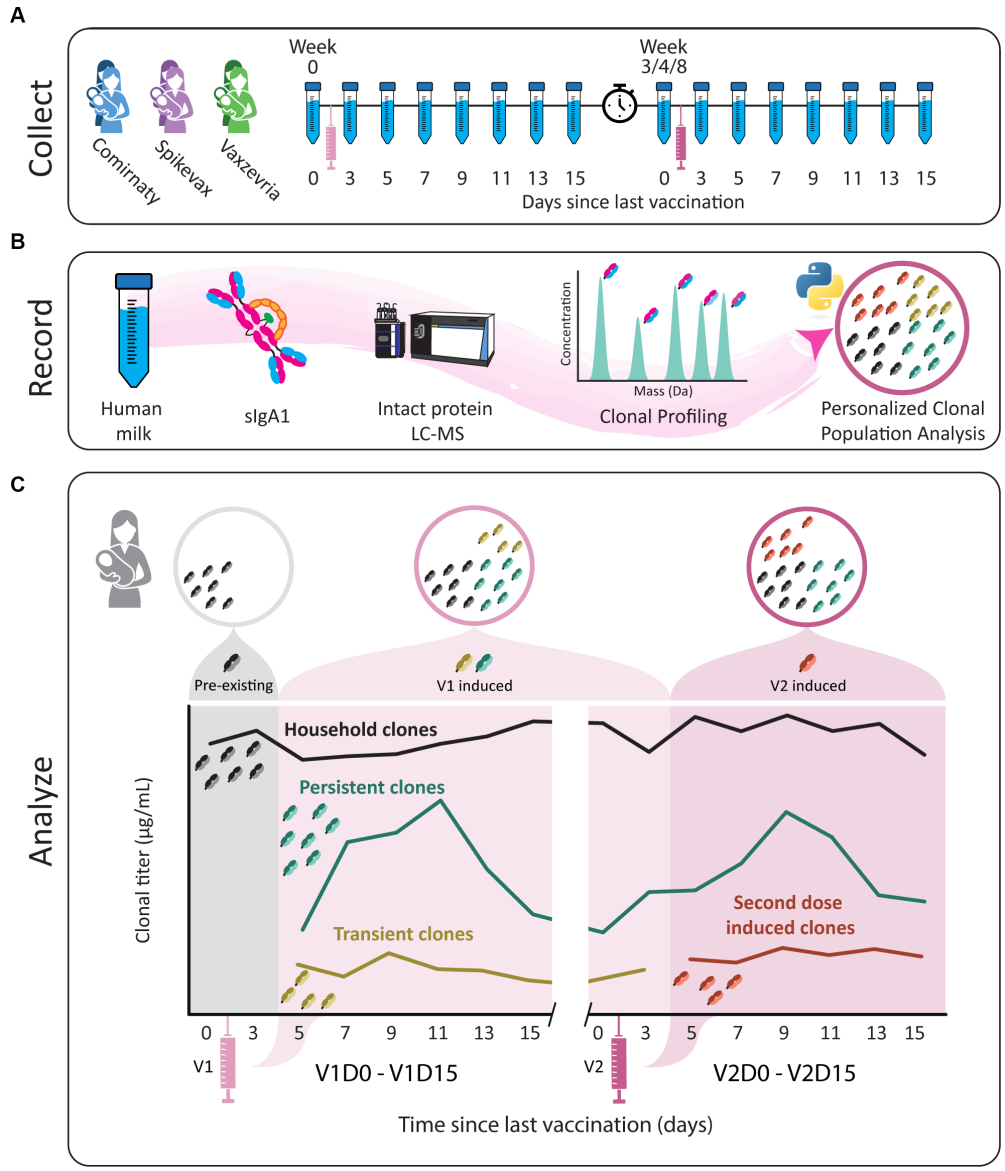
Study workflow. **(A)** Human milk samples were obtained from six donors across 16 timepoints, from just prior to the first vaccination until 15 days after the second vaccination. Individual donors received one of three vaccines, BNT162b2/Comirnaty (blue), mRNA-1273/Spikevax (purple) or AZD1222/Vaxzevria (green). The sample collections are indicated by the tubes and each vaccination with a syringe. The clock indicates the gap in time between vaccinations. **(B)** SIgA1 was affinity-purified from human milk. Subsequently, proteolytically formed SIgA1 Fab fragments were separated and analyzed by LC-MS to obtain a list of clones (i.e., Fab molecules with a unique mass/retention time pair). The concentration of each clone was retrieved at the sampled timepoints using two recombinant IgA internal standards. Clones were then assigned to populations based on their window of detection relative to vaccination, and these populations were analyzed for each donor individually. **(C)** Illustrative examples of abundance profiles of clonal populations over time. The y-axis shows the clonal titer (i.e., the summed concentrations of the clones) for each population over time. Timepoints are referred to as for example V1D3, where D3 indicates the number of days since the last vaccination and V1 indicates the last vaccination. Clones were assigned to one of four populations based on their detection window relative to vaccination. The black line represents *household* clones, SIgA1 clones that were detected in one of the first two timepoints, before a response to vaccination could be expected based on analysis of the parent cohort. All other clonal population were absent from these time points and are considered vaccine induced clones. The remaining three populations designated are *persistent* (teal), *transient* (mustard) and *second dose induced* (maroon) clones. The transient population consists of clones that are only detected in the window V1D5 - V2D3. The persistent clones are clones that arise in the window V1D5 - V2D3 and are also detected after V2D3. Clones in the second dose induced population are clones that were not observed until after V2D3.

**Figure 2:**
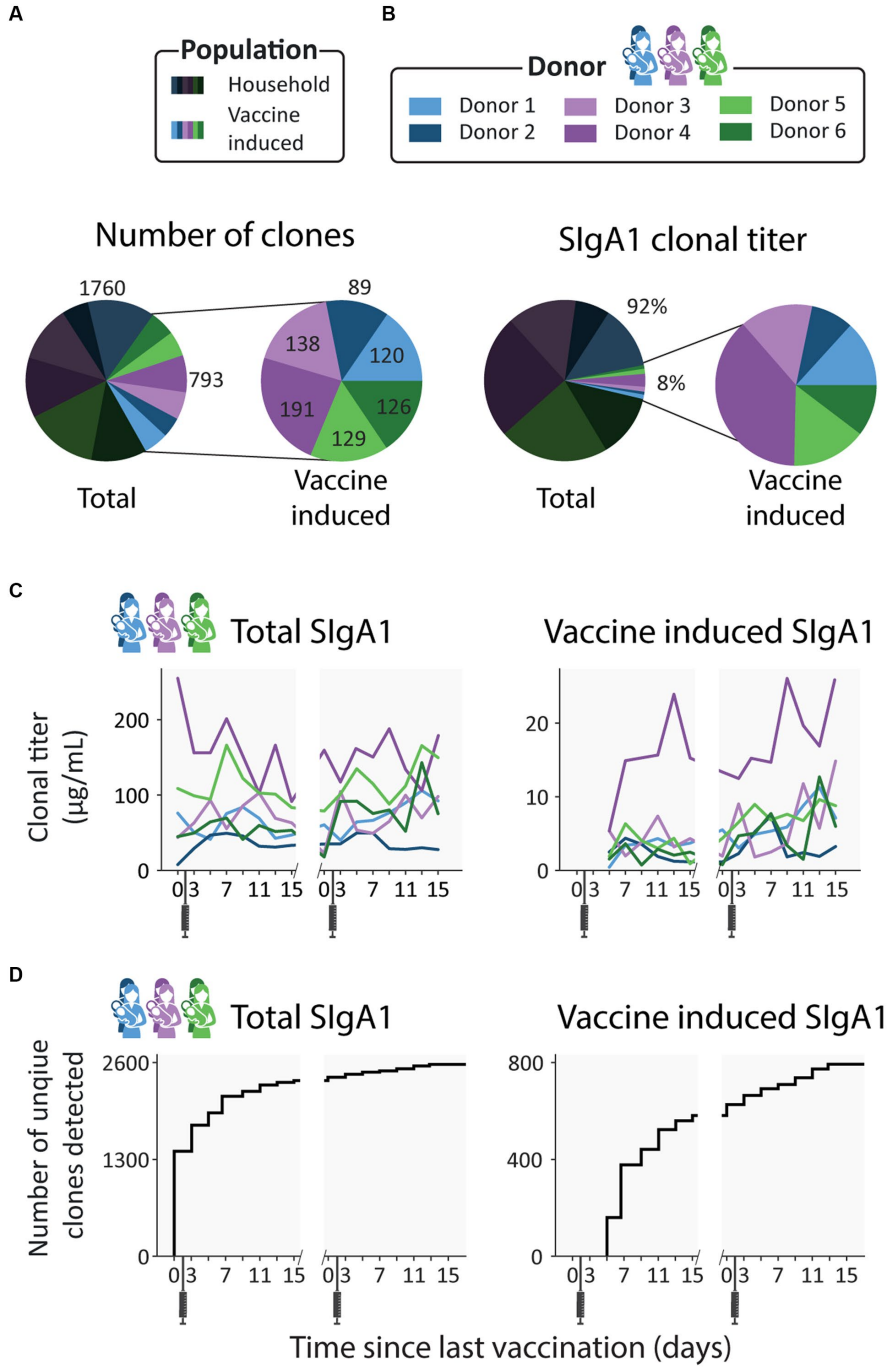
Emergence of novel clones after vaccination. **(A)** Pie charts showing the number of unique clones designated as household (dark) and vaccine induced (light), colored per donor. Vaccine induced clones made up 31% of all detected clones (793 out of 2553 clones). **(B)** Pie charts showing the percent total abundance the household clones (dark) and vaccine induced clones (light), colored per donor. **(C)** Longitudinal changes in total SIgA1 clonal titer (left) and vaccine induced SIgA1 clonal titers (right) for each donor. Vaccine induced SIgA1 clonal titers rise in response to the repeated vaccinations and make up an average of 7% of the total SIgA1 clonal titer. **(D)** Total number of unique clones detected over time (left) and number of unique vaccine induced clones (right). Novel clones emerge shortly after vaccination and by day 7 nearly half of all vaccine induced clones (377 out of 793) have been detected.

In this observational longitudinal case series, we recorded the human milk SIgA1 clonal profiles of six individual donors receiving either Comirnaty, Spikevax or Vaxzevria vaccines (Figure 1; Supplementary Table S1). We detected a total of 2,553 clones across all donors, ranging between 229 and 505 unique clones per donor (Figure 2A), excluding clones that were only found at a single timepoint from all subsequent analysis to limit false discoveries. In line with our previous studies, there was virtually no overlap in the clonal repertoire between donors (only a single clone had an overlapping mass and retention time between donors). In contrast, overlap within each individual donor over the longitudinal sampling was exceptionally high (over 95% of clones were detected at more than one timepoint).

For all donors, the SIgA1 clonal repertoire was dominated by abundant clones that were already detected in the first (V1D0) or second (V1D3) milk sample, before a clonal response is expected as it is prior to or too close to vaccination, as also confirmed based on ELISA data (28) (Supplementary Figure S1). These clones, which we term *household* clones (Figure 1C; Supplementary Figure S2), accounted for 92% of the total SIgA1 *clonal titer* (the summed abundance of all clones) of all samples combined (Figure 2B) and 83–99% of the total SIgA1 clonal titer in any single sample. In each donor, we detected between 89 and 191 clones that emerged more than 3 days after the first vaccination (V1D5 and later) (Figure 2A). These clones, which we termed *vaccine induced* clones (Figure 1C; Supplementary Figure S2), made up 31% of the total detected clones (793 out of 2,553 clones, Figure 2A). These vaccine induced clones were comparatively low in abundance and made up a relatively small portion of the total SIgA1 clonal titer per sample (Figure 2C). Most of the vaccine induced clones emerged shortly after the first vaccination was administered: 47% of the vaccine induced clones (377 clones), were first observed between V1D5 and V1D7 (Figure 2D). This agreed with the ELISA findings for these same samples, where anti-spike SIgA titers started rising around day 5, and further sharp increases were observed 9 days after vaccination (Supplementary Figure S1).

### Novel clonal populations emerge after the second vaccination in all donors

As the vaccines the donors received consist of two doses, we defined four clonal populations based on the window of detection relative to both vaccinations (Figure 1C; Supplementary Table S1). The first population we termed above as *household* clones. These are SIgA1 clones that were detected before a clonal response was expected, at V1D0 or V1D3. The previously described *vaccine induced* clones were categorized into three distinct populations: *transient*, *persistent*, and *second dose induced* clones. The transient and persistent populations are both made up of clones that were detected before timepoint V2D3 but were absent at the first two timepoints (V1D0 and V1D3). The transient clones were *only* detected in the time window from V1D5 to V2D3. Persistent clones arose in this same time window but were also detected after V2D3. Clones in the second dose induced population are clones that were first observed after V2D3.

**Figure 3:**
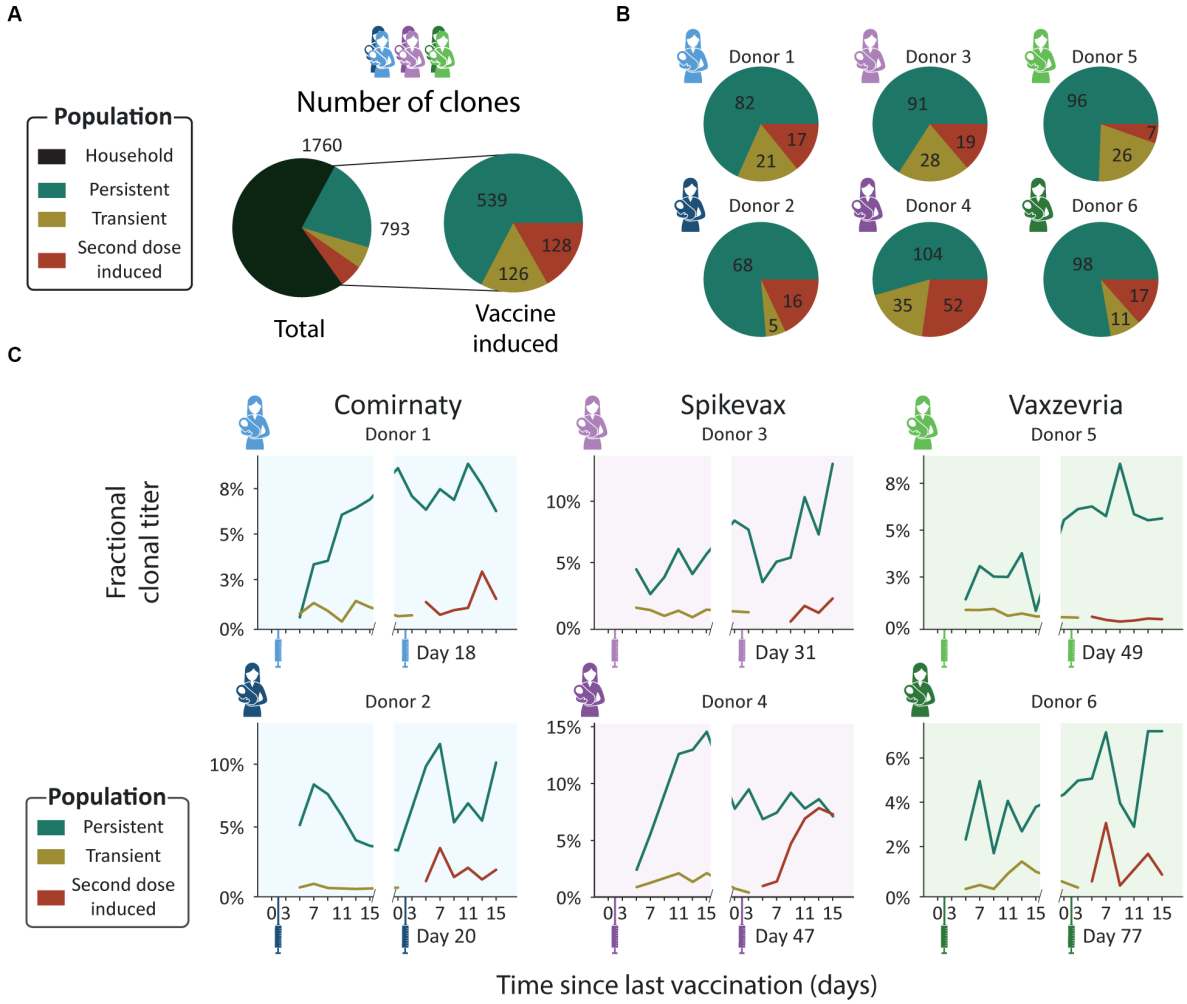
Clonal population analysis. **(A)** Pie charts showing the total number of unique clones in each population. Clones were assigned to populations based on their detection window relative to the vaccination moments: persistent (teal), transient (mustard), and second dose induced clones (maroon). **(B)** Pie charts showing the number of unique clones in each population per donor. Each pie chart shows data for a single donor [Comirnaty (2 blue donors), Spikevax (2 purple donors) and Vaxzevria (2 green donors)]. **(C)** Longitudinal changes in fractional clonal titer (i.e., fraction of the total clonal titer made up by each population) for the vaccine induced clonal populations. Vaccination moments are depicted as color-coded syringes. Each panel shows donor-specific, fractional clonal titers for the three vaccine induced populations. While all donors show a unique repertoire without overlapping clones, varying in number of clones and total clonal titer, when grouped into populations the responses are more consistent. Persistent clones make up the bulk of the vaccine induced SIgA1 clonal titer at nearly every timepoint. The clonal titers of the transient and second dose induced populations account for a much smaller fraction of SIgA1.

These four populations were observed in all donors. Persistent clones were the largest population: 21% of all detected clones were persistent clones (539 clones, Figure 3A), and persistent clones made up between 50 and 80% of donor specific vaccine induced clones (Figure 3B). The transient and second dose induced populations were much smaller and more diverse. The transient and second dose induced populations each make up 5% of all clones (126 and 128 clones respectively, Figure 3A), and 5–20% and 5–27% of donor specific vaccine induced clones (Figure 3B) respectively. When looking at the fractional clonal titer (i.e., the proportion a population contributed to the total SIgA1 clonal titer at a single timepoint) over time, the behavior of these populations was remarkably similar between donors (Figure 3C). The persistent clones dominated here too, as they made up the bulk of the vaccine induced clonal titer at nearly all timepoints and on average of 5.9% of the total SIgA1 clonal titer. Transient and second dose induced populations accounted for a much smaller fraction (on average 0.7 and 1.7% respectively) of the total SIgA1 clonal titer for any single sample (Figure 3C).

### Clonal titer fluctuations can be driven by highly divergent clonal populations

From the ELISA analysis by Juncker et al. (28), donor 4 was identified as the strongest responder in terms of spike-specific IgA. This prompted us to have a closer look at this donor. Our analysis confirmed the strong response, as the vaccine induced clonal titer reached a peak concentration of 26.3 μg/mL, higher than any other donor (maximum 14.7 μg/mL, Figure 2C), and featured 191 unique clones, more than any other donor (maximum 138 clones, Figure 2A).

**Figure 4:**
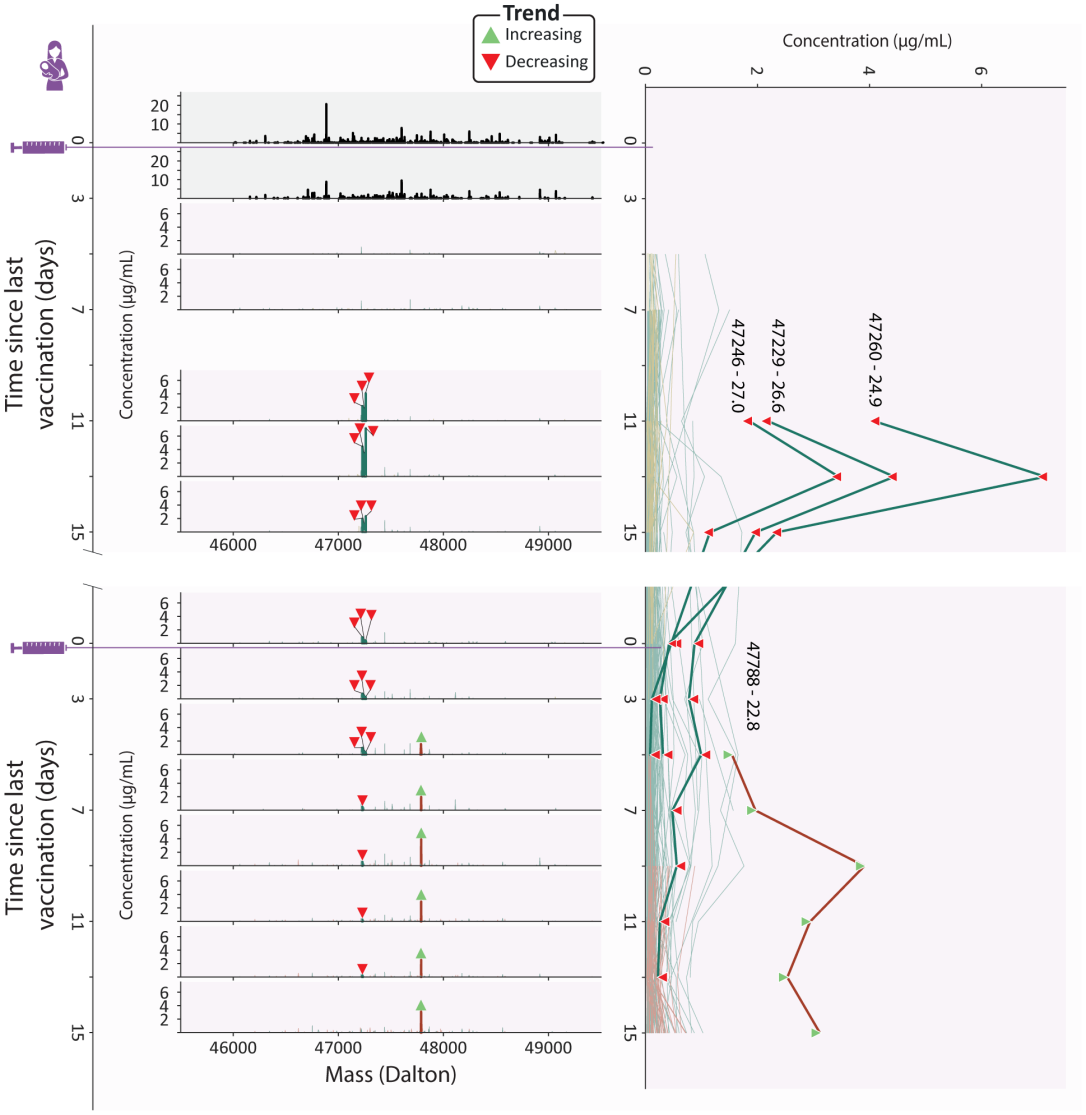
Clonal profile analysis for donor 4, a strong responder. Changes in the vaccine induced clonal profile for donor 4 are depicted, with the 4 most abundant vaccine induced clones annotated by their mass and retention time, highlighted in bold, with each timepoint annotated with a triangle indicating if the clone trends upwards or downwards in concentration over the course of this study. On the left we show mass profiles (SIgA1 clonal concentration in μg/mL) showing either household clones (top two profiles, in black) or vaccine induced clones [subsequent profiles, with individual clones colored according to their assigned population (persistent (teal), transient (mustard), and second dose induced clones (maroon))]. Each peak indicates a single clone and each row a single timepoint. The line plot on the right shows the abundance of individual vaccine induced SIgA1 clones over all timepoints, colored by their population, with the same clones as the mass plots highlighted in bold, labeled with their mass and retention times and annotated with triangles indicating if the clone trends upwards or downwards in concentration throughout the study duration. The highlighted persistent clones are initially highly abundant, but their abundance decreases rapidly, and at the final timepoint none of them are detected. The highlighted second dose induced clone is part of a large and abundant population of second dose induced clones, which at the final timepoints make up 45-50% of the vaccine induced clonal titer.

Uniquely in donor 4, we observed that the second dose induced clonal titer increased comparably to the persistent clonal titer (Figure 3C), indicating that the clonal makeup of the response to the second vaccination was strongly divergent from the response to the initial vaccination. Despite looking very similar to the first phase of the biphasic response (Figure 2C), the second phase of the response was largely driven by the second dose induced population and not the persistent population that was induced by the first dose as the persistent population clonal titer remained relatively stable (Figure 3C). The second dose induced population that drives this second peak in the vaccine induced titer is the largest and most abundant in this study, consisting of 52 unique clones (Figure 3), peaking at over 10 μg/mL (Supplementary Figure S3). Additionally, the second dose induced population made up 45–50% of the vaccine induced clonal titer and 39–44% of vaccine induced clones during the last 3 timepoints (Figure 4), demonstrating how seemingly similar titer fluctuations can be driven by highly divergent clonal populations.

At these final timepoints, the persistent clonal titer had decreased to approximately half its peak value (Supplementary Figure S3). However, we did not observe a similar decrease in the number of detected persistent clones suggesting either a simultaneous drop in the intensity of the individual persistent clones or a strong decrease in abundance of one or more dominant clones from this population. Inspection of the individual persistent clones revealed that at its peak (V1D13), the persistent population included three highly abundant clones which together made up 60% of the persistent clonal titer (Figure 4). While initially these highly abundant clones almost completely dictated the persistent clonal titer fluctuations, they quickly declined in abundance after an initial peak and eventually disappeared while the persistent clonal titer remained relatively stable (Figure 4; Supplementary Figure S2), seemingly causing the persistent clonal titer drop between the first and second phase.

A similarly dominant second dose induced clone was observed to increase in concentration as the three dominant persistent clones were decreasing at V2D5 (Figure 4). The abundance profile of this clone mirrored the upward trending second dose induced titer (Supplementary Figure S3) and was the most abundant clone at the final timepoint, at 3.1 μg/mL (Figure 4). However, 37 other second dose induced clones are still detected at the last timepoint and as we saw with the persistent clones, it may be these lower abundant clones that persist in the long term.

These dominant clones demonstrate how clonal titers fluctuations can be strongly influenced by a limited number of abundant clones. Sometimes these clones only amplify the behavior of their parent population, as with the highlighted second dose induced clone from donor 4. However, they may paint a misleading picture by masking the cumulative behavior of the remaining, lower abundant, clones in the population. The clonal resolution of the LC–MS based Fab profiling method enables us to zoom in on individual clones and allows us to confirm the presence or absence of factors that drive polyclonal responses.

## Discussion

The current body of knowledge about humoral immunity in response to infections and vaccinations are normally determined by ELISAs for total antigen-specific antibody titers and more recently by screening for antigen-specific B-cells. However, recent work by Wolf et al. (27) shows that the antibody titers are poor markers of the frequency of memory B cells after an infection. Therefore, alternative analyses are needed to assess the basics of humoral immunity. We may gain new insights by uncovering when, how and why specific antibodies come up after an infection or vaccination. A first step in doing this is by monitoring individual clones and extracting patterns from clonal populations, as we demonstrate here.

To date, many studies have been conducted to evaluate the antibody titers in human milk from either SARS-CoV-2 infected or vaccinated women. A recent systematic review by Nicolaidou et al. highlights that many different studies were able to detect SARS-CoV-2 specific IgAs and IgGs in human milk after vaccination, however there were inconsistent results of these studies regarding human milk vaccine induced IgAs and their ability to neutralize the virus (29). A possible explanation for this inconsistency could be due to the discrepancy in distinguishing between IgA and SIgA, as it is known for other vaccines like influenza that SIgAs are important for neonatal protection but require special consideration when analyzed (30). Additionally, like Influenza vaccines, it is possible that stronger antibody responses are elicited in the infant if the vaccine is administered during pregnancy, suggesting that also the type of antibody is important for greater protection (18, 31). To further support this, some studies showed no change in IgA in human milk related to neutralization, but showed an increase in anti-SARS-CoV-2 IgGs in human milk lead to neutralization (29). While some of the studies did find back neutralizing IgAs in human milk, these results were not as conclusive as for neutralizing IgGs. In the review by Nicolaidou et al. it was concluded that even though IgA, specifically SIgA, is the dominant Ig in human milk and mucosal surfaces, vaccination against COVID-19 in lactating mothers lead to a dominant IgG response (29). Nicolaidou et al. suggested that the dominant IgG response and low levels of IgA in human milk from the vaccinated mothers could be due to route of exposure to the viral spike protein, via intramuscular vaccination and that IgA is typically an antibody which is important for initial stages of immune responses (29).

Even though the titer levels and neutralization results for human milk IgA were not as strong as for IgG, six studies also tested infant samples to see if SARS-CoV-2 antibodies could be detected in the breastfed infant of vaccinated mothers (31–36). All together the six studies, investigating different infant bio samples, found detectable levels of SARS-CoV-2 specific antibodies in the infant samples. One study by Narayanaswamy et al. assessed the infant stool samples and found back both Anti-RBD IgGs and IgAs suggesting both antibodies survived infant digestion and provided protection to the infant. One recent study showed the long-lasting importance of SIgA in human milk, as spike-specific SIgAs were found back in the human milk samples of COVID-19 infected women 10 months (37) and 1 year after the initial infection (38).

Beyond the evaluation of antibody titers, in this study we detected a heterogeneous polyclonal response to vaccination, as distinct populations of novel clones emerged in every donor after vaccination. We defined three populations of vaccine induced clones, which can be assigned based on their window of detection relative to vaccination: transient, persistent and second dose induced clones (Figure 1C; Supplementary Figure S2). These populations were not only observed in all donors, but also behaved remarkably similar relative to each other, as the persistent population was dominant both in terms of clonal titer and size, regardless of which vaccine the donor had received.

As the donors in this study had not been exposed to SARS-CoV-2 prior to vaccination, the clonal response to the first vaccination can be considered the primary response: a first generation of antibodies, having undergone little to no somatic hypermutation (39). The dominant persistent clones we observe might be the effective portion of the primary response, which are encoded in long lived plasmablasts or memory B cells that proliferate quickly in response to restimulation with the same antigen whereas the transient population could be the ineffective portion of the primary response.

The response to the second vaccination can then be considered the secondary response. In every donor, we observed a population of novel clones emerge during this secondary response. In at least one donor, donor 4, the secondary response had a strongly divergent clonal makeup from the primary response, as it was not predominantly driven by clones induced upon the first vaccine dose but largely by a completely novel clonal population. These novel clones may be the result of a completely new gene recombination but could also be the result of somatic hypermutation of transient or persistent clones, as even small mutations are likely to cause shifts in mass and retention time. Alternatively, they could be clones that escaped detection during the primary response or clones that were derived from previously undetected clones through somatic hypermutation or isotype switching, as our profiling method in this study was limited to SIgA1. While more extensive sequence information is needed to definitively determine the genetic and cellular origin of circulating clones, the second dose induced clones in this study seemingly did not emerge faster than the transient clones, possibly indicating they are not maturations of the first batch of B cells. However, given the small sample size in this study, we are unable to sufficiently answer these questions.

In the parent study of this cohort, spike specific IgAs were longitudinally monitored by ELISA. A biphasic antibody response to SARS-CoV-2 vaccination was observed for spike-specific IgA in these samples, with an accelerated response after the second vaccination, in line with expectations based on leading theories on humoral immune responses (39). We were able to confirm a number of these findings from our case series analysis of this cohort. From both the ELISA and Fab profiling data, donor 4 could be identified as a strong responder, with an observed biphasic response irrespective of analysis type. These confirmations, however, could only be made qualitatively. Quantitatively, we observed a discrepancy between the reported ELISA and measured clonal titers. We believe there are several factors contributing to these discrepancies. First, the applied ELISA measured IgA1 and IgA2, whereas our profiling method detects only IgA1 (9, 28). Secondly, the ELISA based methods may not be fully optimized for SIgAs whereas we know from our previous work that in our LC–MS based Fab method we detect little to no IgAs that are not secretory (5). Furthermore, the ELISA measured spike-specific IgA titers, using a pre-fusion stabilized variant of the spike protein sequence termed 2P (40). Thus, clones that do not bind to the 2P spike protein variant, but perhaps to other components of the vaccine, will not be detected. Similarly, weak binders may be underrepresented in affinity-based assays. As a recent study showed that low rather than high affinity antibodies delivered greater antibody-mediated receptor activity through increased receptor clustering (41), these low affinity clones may be of particular importance.

To date, it is often thought that highly efficient neutralizing antibodies would perhaps not be among the most abundant clones. However, at the current stage of implementation the most reliable detection and quantitation through LC–MS is limited to relatively abundant clones, and low abundant clones likely exist at concentrations below our limit of detection. One way to study these low abundant clones is through fractionation or purification. While there is value in retaining biological context by minimizing purification, simultaneous analysis of the sample in an enriched form can enable a more targeted look at clones of interest or provide us with contextual information about clones in our sample such as binding affinity. For example, in a recent study van Rijswijck et al. (26) analyzed serum samples of SARS-CoV-2 patients, with and without affinity purification, and combined the results to yield information about the cross-reactivity of individual clones to different SARS-CoV-2 variants of concern. This illustrates how the ability to identify and track clones between samples and experiments can be used to obtain functional information about individual clones, and how we can relate this information back to the original abundance profile. Future applications of LC–MS fab profiling hold the promise of high throughput characterization of antibody repertoires, allowing for a greater understanding of the mechanisms related to antibody mediated immunity and defining immune signatures that predict how an individual will respond to future encounters with similar antigens. We imagine this to have future applications similar to HLA phenotypes for organ transplants or genetic markers for cancer treatment. In addition to defining such “biomarkers” for individual patients, we could identify markers of efficacy for individual clones, potentially enabling the direct identification of potential therapeutic antibodies from polyclonal samples. We believe studies like this pave the way to elucidate the mechanisms involved in mounting an effective antibody response and can lead to future targeted efforts to find potential therapeutic candidates.

## Methods

### Study design

In this observational longitudinal case series we used samples from an existing prospective longitudinal study COVID MILK – POWER MILK (28). All participants were subjected to longitudinal analysis of specific antibodies against the SARS-CoV-2 spike-protein by ELISA and general SIgA1 Fab clonal profiling in human milk after vaccination against COVID-19 with BNT162b2/Comirnaty developed by Pfizer-BioNTech, mRNA-1273/Spikevax developed by Moderna or AZD1222/Vaxzevria developed by Oxford/AstraZeneca. Ethical approval was acquired from an Independent Ethics Committee (2020.425/NL74752.029.20). The study was conducted in accordance with the principles of the declaration of Helsinki and the ICH GCP Guidelines, and the Regulation on Medical Research involving Human subjects.

### Subjects

Details concerning subjects have been extensively described (28), demographic details for the six donors in this case series are presented in Supplementary Table S2 and reported symptoms after each vaccination in Supplementary Table S3. Briefly, lactating women in the Netherlands receiving one of the above-described SARS-CoV-2 vaccines were eligible to participate and were recruited through social media platforms. There were no exclusion criteria. All participants were requested to send their vaccination certificate, including the type of vaccination and lot number. From the larger study a subset of 2 women per vaccine group were selected based on the following criteria: (1) a pre-vaccine milk sample was available, (2) data from an enzyme-linked immunosorbent assay (ELISA) with the SARS-CoV-2 spike protein for human milk SIgA was available and indicated high spike-specific SIgA titers. Of the 6 donors in this case series, none had a SARS-CoV-2 infection prior to vaccination, as confirmed with a negative spike-specific ELISA at V0D0 (28). Written informed consent was obtained from all participants.

### Sample collection

Sample collection was performed between January 2021 and July 2021. Human milk samples were collected longitudinally over a period of up to 95 days (Figure 1; Supplementary Table S1). In this study, 16 samples of human milk were analyzed per lactating woman. These samples were collected according to the following schedule: one sample before the first vaccination and one sample on days 3, 5, 7, 9, 11, 13, and 15 after the first vaccination. This schedule was the same for the second vaccination (Supplementary Table S1). Participants were instructed to empty one breast in the morning, before the first feeding moment, and collect 5 mL of milk after mixing the milk. Participants were requested to store the milk samples in the home freezer. Samples were transported back to the lab on dry ice and remained at −80 until analysis (9, 28).

### Fab clonal profiling from human serum and milk

#### IgA enrichment, capture, and digestion

Methods for IgA1 Fab profiling have previously been extensively detailed (3, 4). Briefly, all IgA was captured using CaptureSelect IgA affinity matrix (Thermo Fisher Scientific). Human milk samples were assumed to contain 0.8 μg/μL SIgA and added to excess amount of bead slurry, PBS, and 200 ng of the monoclonals anti-CD20 mIgA1 (7D8-IgA1) and anti-cMET (5D5v2-IgA1). These monoclonals were used as internal standards for quantification, and were a gift from Genmab (Utrecht, NL). Samples were incubated followed by removal of the flow through, containing all non-IgA human milk components. The samples were then washed several times and IgA was digested overnight with the O-glycopeptidase from *Akkermansia muciniphila*, OgpA (OpeRATOR®, Genovis, Llund, Sweden). Digestion was performed using 40 U SialEXO (a sialidase cocktail to remove sialic acids from the O-glycans) and 40 U of OgpA enzyme, and incubated overnight at 37°C, in an Eppendorf thermal shaker (Eppendorf, The Netherlands). Following overnight digestion with OgpA, Ni-NTA agarose slurry was added to the samples to bind the enzyme and incubated for 30 min. Finally, the flowthrough, containing the IgA1 Fabs, was collected by centrifugation.

#### Fab profiling by LC–MS

The LC–MS and data processing approaches as described by Bondt et al. were applied (3, 4). In short, the collected Fab samples were separated by reversed phase liquid chromatography on a Thermo Scientific Vanquish Flex UHPLC instrument, equipped with a 1 mm x 150 mm MAbPac analytical column, directly coupled to an Orbitrap Fusion Lumos Tribrid mass spectrometer (Thermo Fisher Scientific, San Jose, California, USA). The column preheater and the analytical column chamber were heated to 80°C during chromatographic separation. Fab samples were injected as 10 μL and subsequently separated over a 62 min gradient at a flow rate of 150 μL/min. The gradient elution was achieved using mobile phases A (0.1% HCOOH in Milli-Q HOH) and B (0.1% HCOOH in CH3CN), see previous publications for details (3, 4). The instrument was operating in Intact Protein and “Low Pressure” mode for the acquisition of MS data, with a spray voltage of 3.5 kV set from minute 2 to minute 50 of the gradient. The ion transfer tube temperature was set at 350°C, vaporizer temperature at 100°C, sheath gas flow at 15, auxiliary gas flow at 5, and source-induced dissociation (SID) was set at 15 V. Spectra were recorded with a resolution setting of 7,500 (@ 200 m/z) in MS1. Scans were acquired in the range of 500–4,000 m/z with an AGC target of 250% and a maximum injection time set to 50 ms. For each scan 5 μscans were recorded.

#### IgA1 clonal profiling data analysis

Intact masses were retrieved from the generated RAW files using BioPharmaFinder 3.2 (Thermo Scientific). Deconvolution was performed using the ReSpect algorithm between 5 and 57 min using 0.1 or 0.3 min sliding windows with a 25% offset, a merge tolerance of 30 ppm, and noise rejection set to 95%. The output mass range was set from 10,000 to 100,000 with a target mass of 48,000 and mass tolerance 30 ppm. Charge states between 10 and 60 were included and the Intact Protein peak model was selected.

Further data analysis was performed using Python 3.9.13 (with libraries: Pandas 1.4.4 (42), NumPy 1.21.5 (43), SciPy 1.9.1 (44), Matplotlib 3.5.2 (45) and Seaborn 0.11.2). Masses of the BioPharmaFinder identifications (components) were recalculated using an intensity weighted mean considering only the most intense peaks comprising 90% of the total intensity. Using the mAb standards, the intensity was normalized, a relative mass shift was applied to minimize the mass error and a retention time shift was applied to minimize deviation between runs.

Components between 45 and 53 kDa with the most intense charge state above m/z 1,000 and a score of at least 40 were considered Fab portions of IgA1 clones. The clones in samples of the same donor were matched between runs using average linkage (unweighted pair group method with arithmetic mean UPGMA) L∞ distance hierarchical clustering. Flat clusters were formed based on a cophenetic distance constraint derived from a mass and retention time tolerance which were 2 Da and 1 min, respectively. Clones within a flat cluster were considered identical between runs. Clones that were only detected at a single timepoint within a donor were excluded from the analysis. Clones were assigned to populations according to their detection window relative to vaccination as outlined in Supplementary Figure S2.

### Trial registration

This research project was registered at the Dutch Trial Register on May 1st, 2020, number: NL 8575, https://onderzoekmetmensen.nl/nl/trial/23001.

## Data availability statement

The datasets presented in this study can be found online in the MassIVE repository (https://massive.ucsd.edu), under the accession number MSV000092157.

## Ethics statement

The studies involving humans were approved by Ethics Committee of the Amsterdam University Medical Centre. The studies were conducted in accordance with the local legislation and institutional requirements. The participants provided their written informed consent to participate in this study.

## Author contributions

SG: Data curation, Formal analysis, Visualization, Writing – original draft, Writing – review & editing. Software. AB: Formal analysis, Visualization, Writing – original draft, Writing – review & editing, Investigation, Supervision. DR: Formal analysis, Investigation, Writing – review & editing, Methodology. HJ: Formal analysis, Investigation, Methodology, Writing – review & editing, Data curation. SM: Writing – review & editing. MD: Writing – review & editing, Formal analysis, Investigation, Methodology. MH: Methodology, Writing – review & editing, Data curation, Software, Visualization. BK: Data curation, Methodology, Writing – review & editing, Investigation, Supervision. JBvG: Investigation, Supervision, Writing – review & editing, Conceptualization, Funding acquisition, Resources. AH: Conceptualization, Funding acquisition, Investigation, Resources, Supervision, Writing – review & editing, Writing – original draft. KD: Conceptualization, Funding acquisition, Investigation, Supervision, Writing – original draft, Writing – review & editing, Data curation, Formal analysis, Methodology, Visualization.
