# Supplementary material for: A case series exploring the human milk polyclonal IgA1 response to repeated SARS-CoV-2 vaccinations by LC–MS based fab profiling
Source: Front Nutr. 2024 Jan 15;10:1305086. doi: 10.3389/fnut.2023.1305086 (PMC10822949; doi:10.3389/fnut.2023.1305086)
Supplement: Supplementary file 1 [file Data_Sheet_1.docx]

Tables:

|  | **Day** | **Label** | **Comirnaty** | | **Spikevax** | | **Vaxzevria** | |
| --- | --- | --- | --- | --- | --- | --- | --- | --- |
|  |  |  | **1** | **2** | **3** | **4** | **5** | **6** |
|  | **0** | *V1D0* | 0 (88) | 0 (172) | 0 (62) | 0 (242) | 0 (110) | 0 (156) |
|  |  | **First vaccination moment** | | | | | | |
|  | **3** | *V1D3* | 3 | 3 | 3 | 3 | 3 | 3 |
|  | **5** | *V1D5* | 5 | 5 | 5 | 5 | 5 | 5 |
|  | **7** | *V1D7* | 7 | 7 | 7 | 7 | 7 | 7 |
|  | **9** | *V1D9* | 9 | 9 | 9 |  | 9 | 9 |
|  | **11** | *V1D11* | 11 | 11 | 11 | 11 | 11 | 11 |
|  | **13** | *V1D13* | 13 | 13 | 13 | 13 | 13 | 13 |
|  | **15** | *V1D15* | 17 | 15 | NA | 17 | 17 | 15 |
| ***Suggested time between vaccinations*** | | | ***21*** | | ***28*** | | ***54*** | |
|  | **0** | *V2D0* | 18 | 20 | 31 | NA | 49 | 77 |
|  |  | **Second vaccination moment** | | | | | | |
|  | **3** | *V2D3* | 22 | 24 | 34 | 50 | 52 | 80 |
|  | **5** | *V2D5* | 24 | 26 | 36 | 52 | 54 | 82 |
|  | **7** | *V2D7* | 26 | 28 | 38 | 54 | 56 | 84 |
|  | **9** | *V2D9* | 28 | 30 | 40 | 56 | 58 | 86 |
|  | **11** | *V2D11* | 30 | 32 | 42 | 58 | 60 | 88 |
|  | **13** | *V2D13* | 32 | 34 | 44 | 60 | 62 | 90 |
|  | **15** | *V2D15* | 34 | 36 | 48 | 63 | 66 | 95 |

**Table S1: Sampling schedule for each donor**

Notes: The “Day” column indicates the number of days between the last vaccination and the collection of each sample. The “Label” column indicates the label that is to refer to each sample in this manuscript. These are constructed as follows: for a sample labeled V1D3, D3 indicates the number of days since the last vaccination and V1 indicates the last vaccination received by the donor, so this sample was taken three days after the first vaccination. The number of days between the birth of the infant and V1D0 is given in the first row, between brackets. All samples are representative of mature milk. Samples collected just before vaccination are referred to as day 0 (i.e., V1D0 and V2D0). The actual days samples were collected from each individual donor are indicated as the number of days between the first vaccination and each sample collection. For some samples, the number of days between sample collection and the preceding sample collection deviated slightly from the schedule, these samples were underlined. NA indicates that the sample was collected but a date was not recorded. Blank spaces indicate that no sample was collected. The parent study included a follow-up sample, V2D70, for donor 2, 3 and 4. For donor 4, sample V2D15 was lost, and the follow-up sample was used instead. To unify the number of samples analyzed per donor per population, we excluded the remaining 2 follow-up samples from our analysis.

**Table S2:** **Maternal and infant demographics**

| **Donor number** | **Maternal**  **Age (years)** | **Pre-pregnancy**  **BMI** | **Postpartum BMI** | **BMI**  **Category** | **Gravida,**  **Para** | **Mode of**  **Delivery** | **Gestational Age**  **(weeks + days)** | **Birthweight (g)** | **Infant Sex** |
| --- | --- | --- | --- | --- | --- | --- | --- | --- | --- |
| **1** | 31 | 20.4 | 19.8 | healthy  weight | 1,1 | vaginal | 40+3 | 3130 | female |
| **2** | 34 | 23.8 | 22.0 | healthy  weight | 2,1 | vaginal | 39+0 | 3810 | male |
| **3** | 35 | 24.8 | 24.1 | healthy  weight | 1,1 | cesarean | 39+0 | 3405 | male |
| **4** | 32 | 17.7 | 17.7 | underweight | 1,1 | vaginal | 34+0* | 2232 | male |
| **5** | 35 | 38.7 | 38.0 | obesity | 2, 2 | cesarean | 38+5 | 4200 | male |
| **6** | 31 | 23.5 | 23.5 | healthy  weight | 1,1 | vaginal | 40+0 | 3615 | male |

Notes: Maternal age is in years, BMI calculated as kg/m^2^, BMI category defined as: <18.5 = underweight, 18.5-24.9 = healthy weight, 25-29.9 = overweight, >30 = obesity. Postpartum BMIs were assessed at ~7 months, no differences were observed between pre-pregnancy and postpartum BMI categories. All donors were Dutch and non-smokers.

**Table S3: Maternal self-reported vaccine symptoms**

|  | **Vaccine dose 1** | | | | | | **Vaccine dose 2** | | | | | | | |
| --- | --- | --- | --- | --- | --- | --- | --- | --- | --- | --- | --- | --- | --- | --- |
| **Donor number** | **Pain or swelling** | **Fever** | **Fatigue** | **Headache** | **Muscle pain** | **Other** | | **Pain or swelling** | **Fever** | **Fatigue** | **Headache** | **Muscle pain** | **Other** |  |
| **1** | No | No | No | No | No | No | | No | Yes | No | No | No | Yes |  |
| **2** | No | No | No | No | No | No | | Yes | No | No | No | No | No |  |
| **3** | No | No | No | Yes | No | No | | Yes | Yes | No | No | Yes | No |  |
| **4** | Yes | No | No | Yes | Yes | Yes | | No | Yes | No | No | No | No |  |
| **5** | No | No | No | No | Yes | No | | Yes | No | Yes | No | No | No |  |
| **6** | Yes | Yes | No | Yes | No | Yes | | No | Yes | Yes | No | No | No |  |

Notes: Pain or swelling referred to the injection site after vaccination.

# Supplement Figure Legends


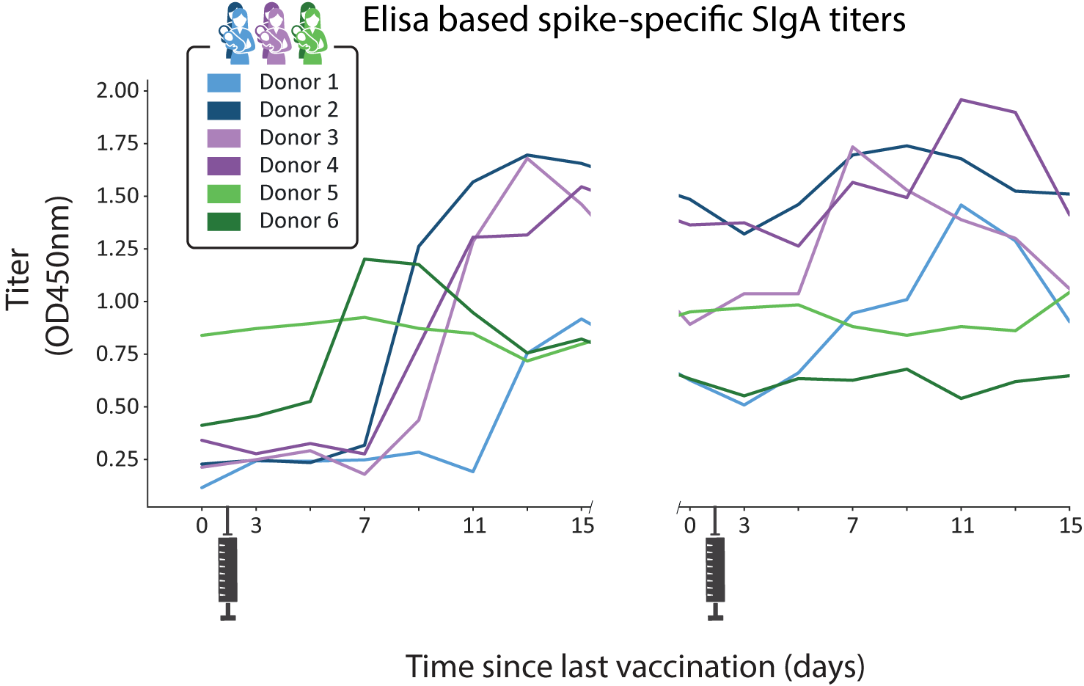


**Figure S1: Quantified ELISA Spike-specific IgA titers**. A biphasic antibody response to SARS-CoV-2 vaccination was observed for spike-specific IgA, with an accelerated response after the second vaccination. Original data from Juncker et al. (1).


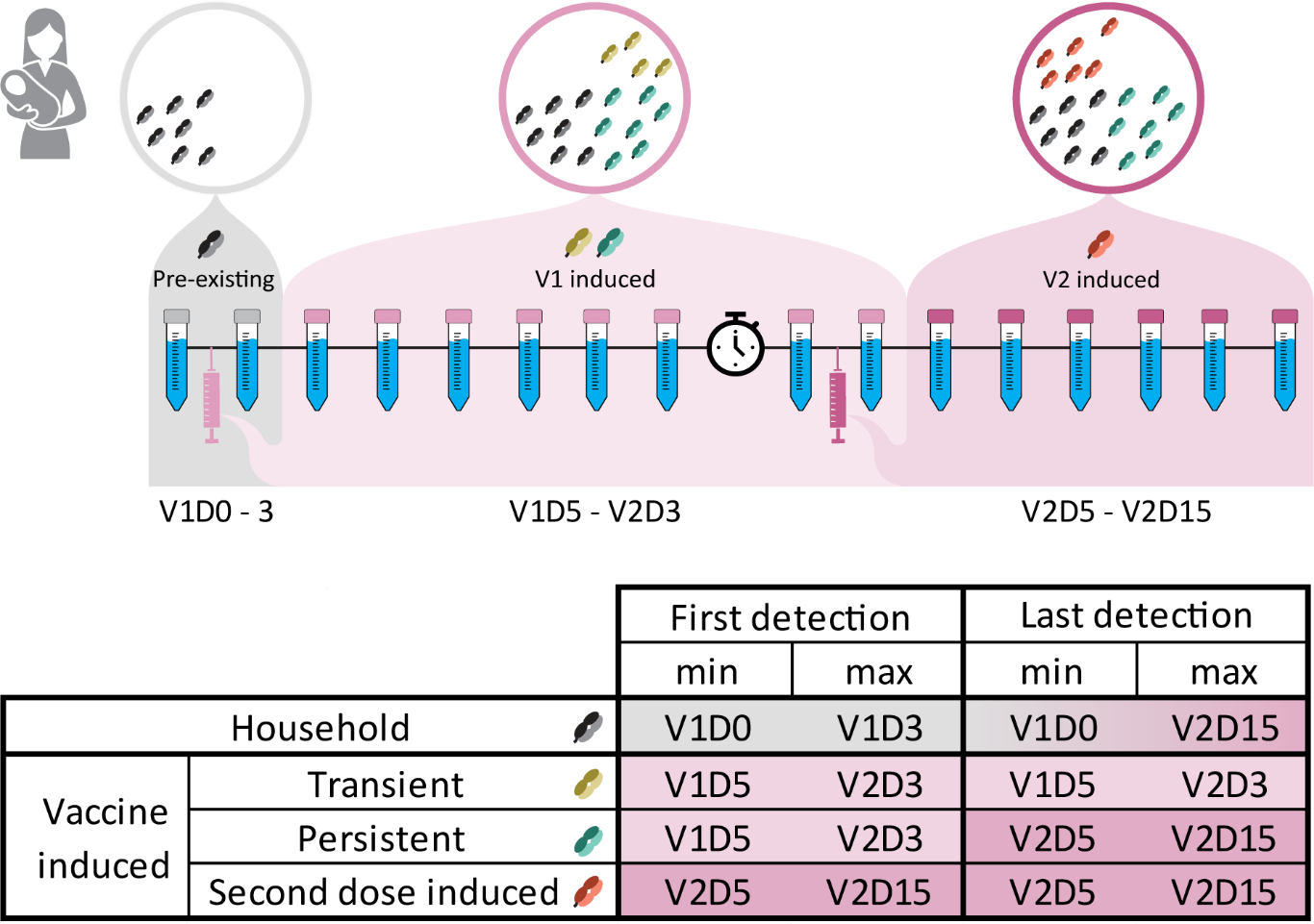


**Figure S2:** **Clonal population inclusion criteria.** Description of the criteria used to assign clones to the designated clonal populations based on their first and last detection moment (i.e., their detection window) relative to vaccination. Time windows are colored by appearance of clones relative to vaccination. Grey window (V1D0 – V1D3): Not attributed to vaccination. Light pink window (V1D5 – V2D3): Attributed to the first vaccination. Dark pink window (V2D5 – V2D15): Attributed to the second vaccination. Clones not detected in the first window were considered vaccine induced clones, and further assigned as follows: Clones first detected in the light pink window were considered transient clones if they were only detected in the light pink window, or persistent clones if they were detected in the dark pink window as well. Clones first detected in the dark pink window were considered second dose induced clones.


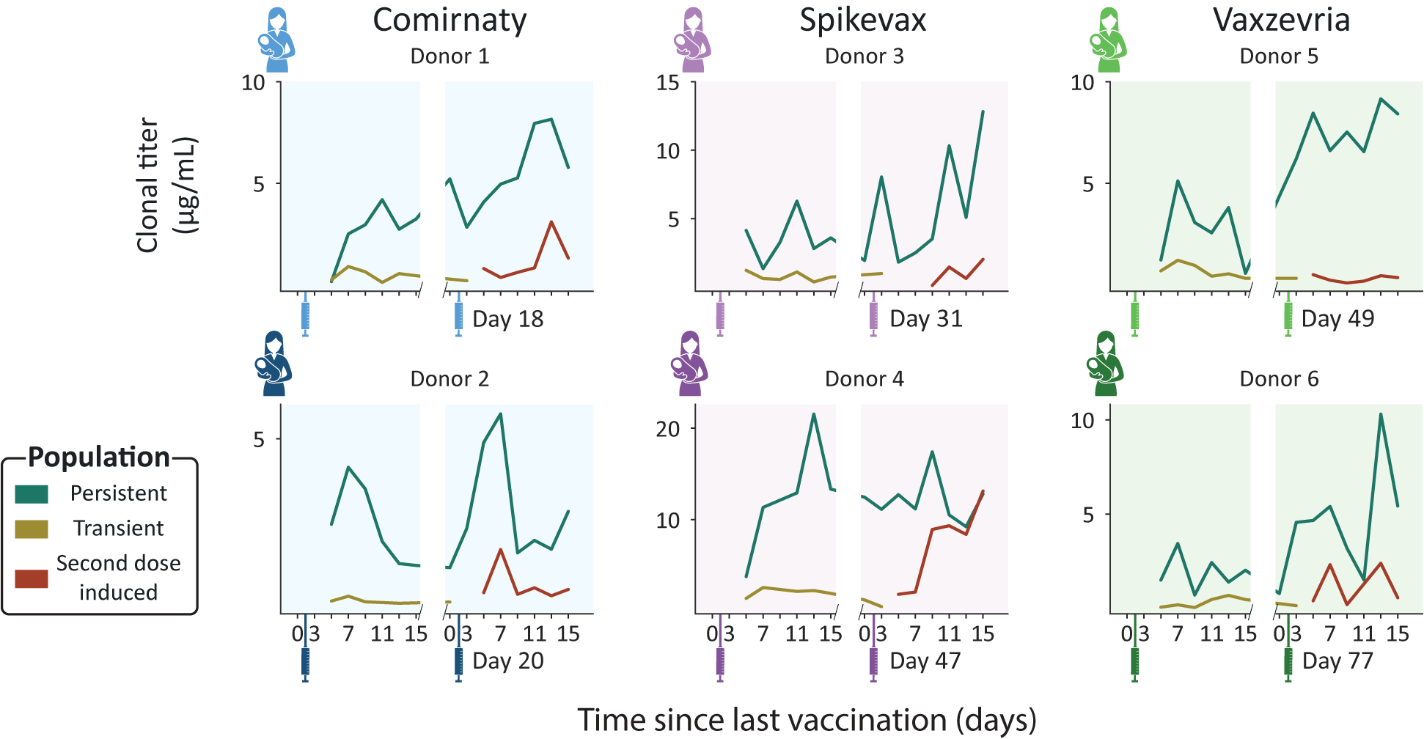


**Figure S3: Longitudinal changes in absolute clonal titers for the vaccine induced populations**. Each panel shows the population clonal titer (i.e., the summed concentrations of the individual SIgA1 clones) for our three assigned, vaccine induced populations: Persistent clones (teal), transient (mustard) and second dose induced (maroon). Each panel shows data for a single donor (Comirnaty (2 blue donors), Spikevax (2 purple donors) and Vaxzevria (2 green donors)). Vaccination moments are depicted as color-coded syringes. Each panel shows donor-specific, clonal titers for the three vaccine induced populations. While all donors show a unique repertoire without overlapping clones, varying in number of clones and total clonal titer, when grouped into populations the responses are more consistent. Persistent clones make up the bulk of the vaccine induced SIgA1 clonal titer at nearly every timepoint. The clonal titers of the transient and second dose induced populations account for a much smaller fraction of SIgA1.

# References

1. Juncker HG, Mulleners SJ, Ruhé EJM, Coenen ERM, Bakker S, van Doesburg M, et al. Comparing the human milk antibody response after vaccination with four COVID-19 vaccines: A prospective, longitudinal cohort study in the Netherlands. EClinicalMedicine (2022) 47. doi: 10.1016/J.ECLINM.2022.101393.
